# The influence of age in the progression from pre-myopia to myopia onset: a 1 year retrospective analysis

**DOI:** 10.3389/fmed.2026.1872078

**Published:** 2026-07-07

**Authors:** Shuang Wang, Keke Huang, Xingyu He, Zhanfeng Wang

**Affiliations:** Department of Ophthalmology, Chengdu Third People’s Hospital, Affiliated Hospital of Southwest Jiaotong University, Chengdu, Sichuan, China

**Keywords:** age, axial elongation, myopia, pre-myopia, spherical equivalent refraction

## Abstract

**Background:**

Pre-myopia is a critical stage before myopia onset, yet little is known about how age influences axial elongation during the transition from pre-myopia to myopia. This study aimed to investigate the effect of age on annual axial elongation during this 1 year conversion period.

**Methods:**

This retrospective study included 53 children (53 right eyes) aged 6–10 years who progressed from pre-myopia (spherical equivalent refraction ≤ + 0.75 D and > − 0.50 D) to myopia (≤ − 0.50 D) within 1 year. Annual axial elongation (ΔAL) was calculated as the absolute change in axial length over 1 year. Analysis of covariance (ANCOVA) was performed with ΔAL as the dependent variable, age group (6–9 years) as a fixed factor, and baseline axial length and baseline spherical equivalent refraction as covariates. Pairwise comparisons were adjusted using the Bonferroni method. Age was further dichotomized into 6–7 years and 8–9 years for subgroup analysis, and also analyzed as a continuous variable using multiple linear regression.

**Results:**

Annual axial elongation decreased with increasing age: 0.76 ± 0.16 mm (6y), 0.64 ± 0.17 mm (7y), 0.54 ± 0.24 mm (8y), 0.37 ± 0.14 mm (9y), and 0.38 ± 0.03 mm (10y). After adjusting for baseline ocular biometrics, ANCOVA revealed a significant main effect of age on ΔAL [*F*(3, 45) = 3.689, *p* = 0.019, partial η^2^ = 0.197]. Bonferroni-corrected comparisons showed that children aged 6 and 7 years exhibited significantly greater axial elongation than those aged 9 years (mean differences = 0.391 mm and 0.271 mm, respectively; both *p* < 0.01). In the dichotomized comparison, the 6–7-year group had significantly greater ΔAL than the 8–9-year group (0.653 mm vs. 0.499 mm, *p* = 0.009). When analyzed as a continuous predictor, each additional year of age was associated with a 0.093 mm decrease in annual axial elongation [*β* = −0.093, 95% CI (−0.150, −0.035), *p* = 0.002].

**Conclusion:**

Among children who converted from pre-myopia to myopia within 1 year, younger age was associated with greater axial elongation. These findings highlight the importance of early surveillance and preventive strategies in the pre-myopic stage, particularly for children aged 6–7 years. Larger prospective studies controlling for environmental and familial risk factors are needed.

## 1 Introduction

Myopia has become a global public health issue: approximately 50% of the populations of several industrialized countries have myopia (1), and it is estimated that by 2050, nearly 4.8 billion people worldwide will be affected by myopia (2). Myopia not only leads to low vision but is also a risk factor for several eye diseases, such as cataract, glaucoma, retinal detachment, and macular degeneration (3). It is also one of the leading causes of blindness globally (4).

Pre-myopia, in addition to myopia, is receiving increasing attention. According to the definition proposed by the International Myopia Institute (IMI) in 2019 (5), “pre-myopia” refers to a refractive state in children where the spherical equivalent is ≤ + 0.75 D and > − 0.50 D. Much research (6) has focused on how to slow myopia progression after the onset, yet studies on the transition from pre-myopia to the onset of myopia are less.

Research by Xiang et al. (7) indicated that the rate of spherical equivalent refraction (SER) progression was the highest during the year of onset. After the first detection of myopia, the rate of SER progression decreased in the following year and kept decreasing. Annual change in axial length showed a similar result. He’s another research (8) showed that the younger the age at myopia onset, the greater the likelihood of progressing to high myopia. Among children with onset at 7–8 years of age, 53.9% progressed to high myopia in adulthood. However, those with onset at age 9, only 32.4% developed high myopia; for onset at age 10, the figure dropped to just 19.4%. The two studies above revealed the important role of age in the onset and development of myopia and that younger age at onset predicts high myopia.

However, few studies have specifically examined axial elongation during the 1 year transition period from pre-myopia to myopia. This gap is clinically significant because accelerated axial growth during the conversion year may be the biological mechanism linking younger age to earlier onset and greater risk of high myopia. We therefore conducted a retrospective study to investigate how age influences the rate of axial elongation during the 1 year transition from pre-myopia to myopia, using data from children who all progressed from a pre-myopic state to myopia within 1 year.

## 2 Materials and methods

### 2.1 Study design

In this retrospective study, we collected the data of school-age children (aged, 6–12 years) with the onset of myopia who were diagnosed at the Chengdu Third People’s Hospital in Chengdu, China, from January 2022 to August 2025. The patients’ data that were collected included their age, sex, spherical equivalent refraction (SER) and axial length (AL). The patients were then categorized into age groups (6, 7, 8, 9, and 10 years). Each patient had regular follow-ups every 6 months, and data were recorded for a period of 1 year. To account for the correlation between two eyes of the same subject, only data from the right eye were included in the final statistical analysis. All consecutive eligible patients were enrolled. No *a priori* sample size calculation was performed.

### 2.2 Inclusion and exclusion criteria

The inclusion criteria were as follows: (1) initial age of the child between 6 and 12 years; and (2) The initial spherical equivalent refraction ranged from ≤ + 0.75 D and > − 0.50 D, indicating pre-myopia; 1 year later, the spherical equivalent refraction ≤ − 0.50 D signified myopia onset.

The exclusion criteria were as follows: (1) the presence of any systemic diseases affecting vision; (2) other diagnosed ophthalmic diseases, including congenital cataracts, retinal detachment, retinoblastoma, keratoconus, etc.; (3) exophoria of more than 3 prism diopters, intermittent exotropia, and concomitant exotropia, as well as other forms of strabismus; (4) refractive disparity (≥1.00 D), (5) early-onset myopia occurring within 6 years of age; and (6) history of myopia intervention, including low-intensity repeated red light therapy, low-dose atropine, 2 h of outdoor activity daily, or wearing specially designed lenses.

### 2.3 Examination items and criteria

#### 2.3.1 Cycloplegic refraction

All children were administered one drop per eye of topical tropicamide eye drops (0.5%, manufactured by Shandong Bausch & Lomb FruiTech Pharmaceuticals Co. Ltd., China), and this was repeated six times at 5-min intervals. After cycloplegia, retinoscopy was performed, followed by subjective refraction. Refraction was performed 30 min after the six instillations. Absence of light reflex and pupil size were confirmed before examination. Cyclopentolate was unavailable at our institution and was therefore not used. The same protocol was applied at both baseline and follow-up. All refractions were recorded using the spherical equivalent refraction (SER).

#### 2.3.2 Axial length measurement

Axial length measurements were conducted using the IOLMASTER700 (produced by Carl Zeiss Meditec, Germany), and the data collected were recorded to two decimal places.

### 2.4 Statistical analysis

ANCOVA was conducted to evaluate the effect of age on 1 year right-eye axial length elongation (ΔAL), while statistically controlling for potential confounding effects of baseline ocular biometrics. A total of 51 children were categorized into four age groups (6, 7, 8, and 9 years; the 10-year group was excluded due to small sample size, *n* = 2), and the analysis utilized right-eye measurements exclusively. The dependent variable was defined as the absolute change in axial length over 1 year (ΔAL = 1-year follow-up AL − baseline AL, in millimeters). Age group was entered as a fixed between-subjects factor with four levels. Two continuous covariates were included in the model to adjust for baseline differences across age groups: baseline right-eye axial length (baseline AL, mm) and baseline right-eye spherical equivalent refraction (baseline SER, diopters), both of which are established determinants of subsequent axial elongation in pediatric populations. The full ANCOVA model was specified as ΔAL ~ Age Group + Baseline AL + Baseline SER, employing Type II sums of squares for hypothesis testing. Given that age group was the primary predictor of interest, the main effect of age was evaluated first; if statistically significant, *post hoc* pairwise comparisons between all six possible age group pairs were performed using the Bonferroni adjustment to control the family-wise error rate, with an adjusted significance threshold of *α* = 0.0083 (0.05/6). Effect sizes were reported as partial eta-squared (partial η^2^) for each model term. Prior to conducting ANCOVA, the underlying statistical assumptions were systematically verified: (a) normality of ΔAL within each age group was assessed using the Shapiro–Wilk test; (b) homogeneity of variance across groups was evaluated using Levene’s test; and (c) the homogeneity of regression slopes assumption was tested by fitting a full factorial model including all two-way interactions between age group and each covariate (Age Group × Baseline AL, Age Group × Baseline SER); if interaction terms were non-significant, the reduced main-effects model was deemed appropriate. Statistical significance was set at *α* = 0.05 for two-tailed tests. All analyses were performed using Python 3.x with the statsmodels library.

**Figure 1:**
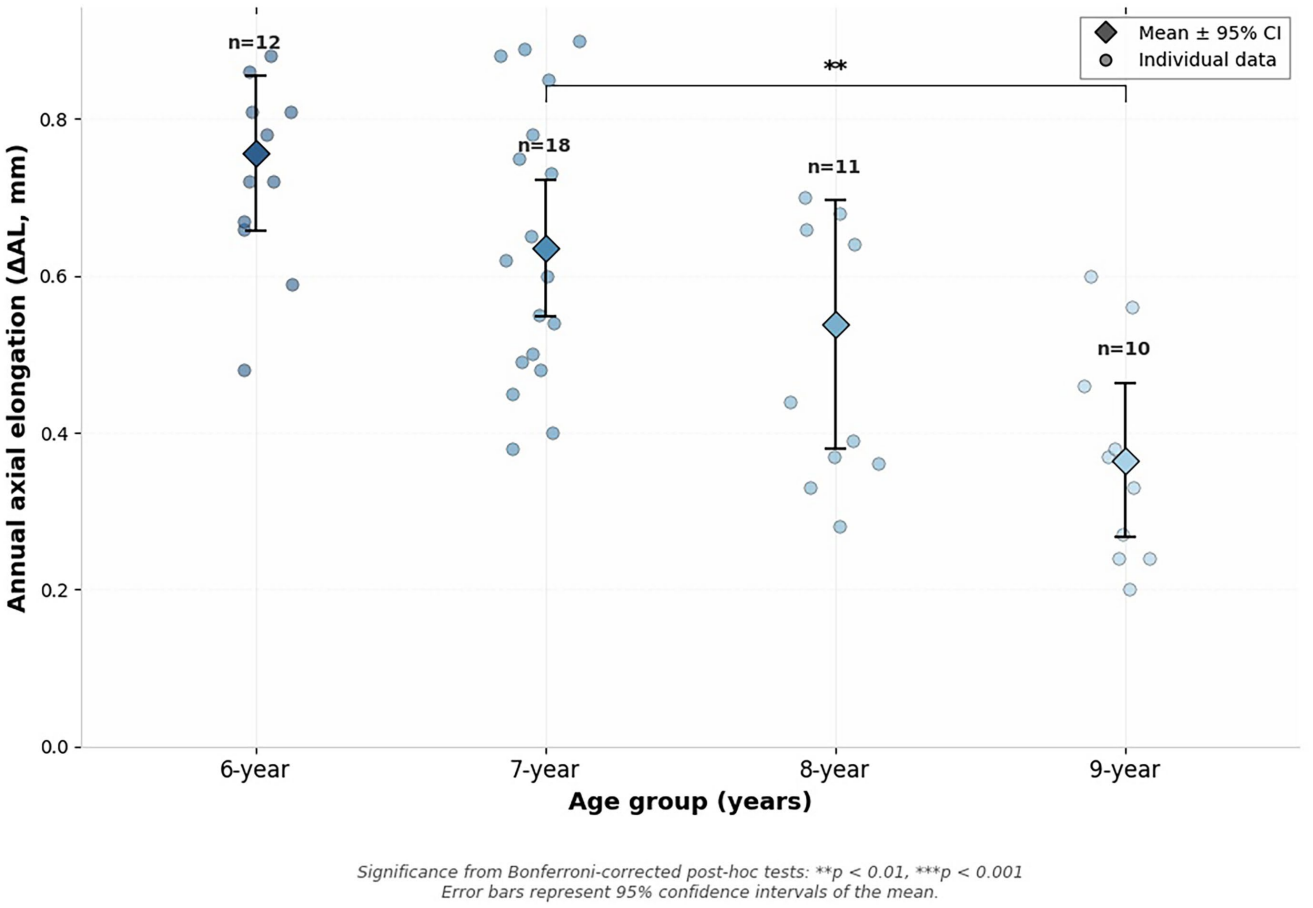
Annual axial elongation (ΔAL) of the right eye over 1 year across age groups (6–9 years). Individual data points are shown as circles, and the diamond markers represent group means with 95% confidence intervals (CIs). Sample sizes are indicated as n. The mean ΔAL values were 0.76 mm (95% CI: 0.66–0.85) for the 6-year group, 0.64 mm (95% CI: 0.55–0.72) for the 7-year group, 0.54 mm (95% CI: 0.38–0.70) for the 8-year group, and 0.37 mm (95% CI: 0.27–0.46) for the 9-year group. Analysis of covariance (ANCOVA) revealed a significant main effect of age on ΔAL after controlling for baseline axial length and baseline spherical equivalent refraction [*F*(3, 45) = 3.689, *p* = 0.019]. Bonferroni-corrected *post hoc* pairwise comparisons showed that the 6-year group differed significantly from the 9-year group (mean difference = 0.391 mm, *p* < 0.001), and the 7-year group differed significantly from the 9-year group (mean difference = 0.271 mm, *p* = 0.001). All other pairwise comparisons were non-significant after Bonferroni correction (all *p* > 0.05). **p < 0.01, ***p < 0.001.

In addition to categorical analysis, age was also analyzed as a continuous variable using multiple linear regression with ΔAL as the dependent variable and age (in years), baseline AL, and baseline SER as independent predictors. Age was entered as a continuous predictor (in years), sex as a binary variable (0 = female, 1 = male), and baseline axial length (AL) and baseline spherical equivalent refraction (SER) were included as covariates to control for baseline ocular biometrics. An age × sex interaction term was also tested to evaluate whether the effect of age on ΔAL differed between boys and girls. Prior to analysis, multicollinearity was assessed using variance inflation factors (VIF); all predictor VIFs were below 2, indicating no severe multicollinearity. Model residuals were examined for normality using the Shapiro–Wilk test. Model comparison was performed using nested F-tests to evaluate the contribution of sex and the age × sex interaction. The final model was selected based on adjusted *R*^2^, Akaike information criterion (AIC), and the principle of parsimony (see Figure 1).

## 3 Results

### 3.1 Participants general characteristics

A total of 53 children [30 male children (56.60%) and 23 female children (43.40%)] from pre-myopia to myopia (choosing the right eye including 53 eyes), aged between 6 and 10 years (mean age, 7.47 ± 1.53 years) were included in this study. The initial axial length (AL) was 23.32 ± 0.69 mm, and the final axial length (AL2) was 23.89 ± 0.61 mm. The 1-year AL elongation (△AL) was 0.58 ± 0.22 mm.

The distribution across groups was as follows: 6-year group: 12 cases (12 eyes); 7-year group: 18 cases (18 eyes); 8-year group: 11 cases (11 eyes); 9-year group: 10 cases (10 eyes); 10-year group: 2 cases (2 eyes). The basic characteristics of the groups are listed in Table 1.

**Table 1:** Baseline data of the group and age subgroup.

Annual axial elongation by age group in children progressing to myopia	Main group	6-year group	7-year group	8-year group	9-year group	10-year group
	*n* = 53	*n* = 12	*n* = 18	*n* = 11	*n* = 10	*n* = 2
Eye number	53	12	18	11	10	2
Age (years)	7.47 ± 1.53	6	7	8	9	10
Sex, male/female	30/23	4/8	13/5	7/4	5/5	1/1
Initial Spherical equivalent refraction, SER1 (D)	+0.08 ± 0.34	+0.44 ± 0.32	+0.06 ± 0.29	+0.05 ± 0.24	−0.12 ± 0.24	−0.25 ± 0.00
Spherical equivalent refraction after 1 year, SER2 (D)	−0.88 ± 0.32	−0.79 ± 0.28	−1.00 ± 0.39	−0.82 ± 0.22	−0.85 ± 0.29	−1.00 ± 00
Spherical equivalent refraction progression (△SER) (D)	−0.97 ± 0.32	−1.23 ± 0.31	−1.06 ± 0.35	−0.86 ± 0.32	−0.73 ± 0.38	−0.75 ± 0.00
Initial axial length (AL1) (mm)	23.32 ± 0.69	22.93 ± 0.58	23.17 ± 0.57	23.44 ± 0.75	23.73 ± 0.57	24.30 ± 0.54
Axial length after 1 year (AL2) (mm)	23.89 ± 0.61	23.69 ± 0.58	23.80 ± 0.61	23.98 ± 0.72	24.10 ± 0.52	24.68 ± 0.51
Axial length elongations (△AL) (mm)	0.58 ± 0.22	0.76 ± 0.16	0.64 ± 0.17	0.54 ± 0.24	0.37 ± 0.14	0.38 ± 0.03

### 3.2 Comparison within the group

Descriptive statistics for all 53 children are presented in Table 1: during the year from pre-myopia to myopia onset, annual axial-length elongation (△AL) slows with increasing age; the mean increases are 0.76 ± 0.16 mm, 0.64 ± 0.17 mm, 0.54 ± 0.24 mm, 0.37 ± 0.14 mm, and 0.38 ± 0.03 mm for ages 6 to 10 years, respectively. Because the 10-year group contained only 2 children, all subsequent inferential analyses were restricted to the 51 children aged 6–9 years.

To examine the effect of age on annual axial elongation (ΔAL), we first conducted an analysis of covariance (ANCOVA) with age group (6/7/8/9 years) as a categorical factor and baseline axial length (AL) and baseline spherical equivalent refraction (SER) as covariates. ANCOVA revealed a significant main effect of age group on ΔAL [*F*(3, 45) = 3.689, *p* = 0.019, partial η^2^ = 0.197], whereas neither baseline AL [*F*(1, 45) = 1.536, *p* = 0.222] nor baseline SER [*F*(1, 45) = 1.727, *p* = 0.195] showed significant independent effects. The overall model explained 42.8% of the variance in ΔAL (*R*^2^ = 0.428, adjusted *R*^2^ = 0.364). Bonferroni-corrected *post hoc* pairwise comparisons indicated that the 6-year group (mean ± SD: 0.756 ± 0.156 mm) and the 7-year group (0.636 ± 0.175 mm) both exhibited significantly greater ΔAL than the 9-year group (0.365 ± 0.138 mm; mean differences = 0.391 mm and 0.271 mm, respectively; both *p* < 0.01); all other pairwise comparisons were non-significant after correction (all *p* > 0.05) (Table 2).

**Table 2:** Comparison of different ages within the age group.

Comparison	Mean difference (mm)	95% CI	*t*-value	df	Uncorrected p	Bonferroni p	Significance
6-year vs. 7-year	+0.120	[−0.005, +0.246]	1.971	25.6	0.0596	0.3579	ns
6-year vs. 8-year	+0.218	[+0.040, +0.395]	2.587	17.1	0.0191	0.1147	ns
6-year vs. 9-year	+0.391	[+0.260, +0.522]	6.237	19.9	0.0000	<0.001	***
7-year vs. 8-year	+0.097	[−0.076, +0.271]	1.185	16.7	0.2527	1.0000	ns
7-year vs. 9-year	+0.271	[+0.146, +0.395]	4.507	22.7	0.0002	<0.001	***
8-year vs. 9-year	+0.173	[−0.003, +0.350]	2.076	16.4	0.0540	0.3238	ns

Pairwise comparisons of annual axial elongation (ΔAL) among age groups. Bonferroni-corrected p-values are reported (six comparisons, adjusted *α* = 0.0083). ns, not significant; *p* < 0.05; *p < 0.01; **p < 0.001.

Given that adjacent age groups did not differ significantly, we further compared a younger group (6–7 years, *n* = 30) with an older group (8–9 years, n = 21) using a two-group ANCOVA. This analysis confirmed that younger children had significantly greater axial elongation than older children [*F*(1, 47) = 7.385, *p* = 0.009], with adjusted marginal means of 0.653 mm versus 0.499 mm at the grand means of the covariates.

We additionally performed a multiple linear regression treating age as a continuous variable and including sex as a covariate. After adjusting for baseline AL, baseline SER, and sex, age was a significant negative predictor of ΔAL [*β* = −0.093, 95% CI (−0.150, −0.035), *t*(46) = −3.227, *p* = 0.002], indicating that each additional year of age was associated with a 0.093 mm decrease in annual axial elongation. Sex did not exhibit a significant main effect (*β* = 0.011, *p* = 0.845), and the age × sex interaction was also non-significant (*β* = −0.076, *p* = 0.134). The continuous-age model explained 42.1% of the variance (*R*^2^ = 0.421, adjusted *R*^2^ = 0.371). Model assumptions were satisfied: all variance inflation factors were below 2, and residuals were normally distributed (Shapiro–Wilk W = 0.969, *p* = 0.209) (see Table 3).

**Table 3:** Baseline and 1-year follow-up data of the 6–7 years group and 8–10 years group.

Parameter	Younger group (6–7 years)	Older group (8–9 years)	*p*-value
Sample size, *n*	30	21	
Sex, male/female	17/13	12/9	1.0000^a^
Initial Spherical equivalent refraction, SER1 (D)	+0.21 ± 0.35	−0.04 ± 0.25	0.0061*
Spherical equivalent refraction after 1 year, SER2 (D)	−0.92 ± 0.36	−0.83 ± 0.25	0.3385
Spherical equivalent refraction progression (△SER) (D)	−1.12 ± 0.34	−0.80 ± 0.35	0.0018*
Initial axial length (AL1) (mm)	+23.07 ± 0.58	+23.58 ± 0.67	0.0080*
Axial length after 1 year (AL2) (mm)	+23.76 ± 0.57	+24.03 ± 0.62	0.1119
Axial length elongations (△AL) (mm)	+0.68 ± 0.18	+0.46 ± 0.21	<0.001*

Comparison of baseline and 1-year follow-up data between the younger group (6–7 years) and the older group (8–9 years). Data are presented as mean ± standard deviation. *p*-values from Welch’s independent-samples *t*-test (continuous variables) or chi-square test (sex); *p* < 0.05.

a Chi-square test.

*Means significant difference.

Taken together, these complementary analyses—categorical ANCOVA, dichotomized group comparison, and continuous multiple regression—converge on a consistent conclusion: axial elongation decelerates progressively with age during childhood, with the most rapid growth occurring in the youngest children (≤7 years), and this pattern is independent of sex and baseline ocular biometrics.

## 4 Discussion

Pre-myopia is an important concept for myopia control. According to our findings, axial elongation has already accelerated markedly in the year preceding the onset of myopia which is similar to Research by Xiang et al. (7). Our study also found that the younger the age, the greater the magnitude of axial elongation from the pre-myopia to myopia. Research by Xiang et al. (7) compared axial-length changes during the year of myopia onset with those seen after myopia had already developed, whereas our study focuses on among children who progressed from pre-myopia to myopia within 1 year, younger age was associated with greater axial elongation.

Our ANCOVA results revealed that age exerted a significant main effect on 1 year axial elongation after controlling for baseline AL and baseline SER [*F*(3, 45) = 3.689, *p* = 0.019], with the youngest children (6–7 years) showing the fastest axial growth and a gradual deceleration across successive age groups. *Post hoc* Bonferroni comparisons confirmed that children aged 6 and 7 years exhibited significantly greater annual ΔAL than those aged 9 years (mean differences = 0.391 mm and 0.271 mm, respectively; both *p* < 0.01), whereas differences between adjacent age groups were not statistically significant. This pattern indicates that axial elongation decelerates progressively with age rather than dropping abruptly at a specific threshold. The finding that baseline AL and baseline SER did not independently predict ΔAL once age was accounted for suggests that the age-related deceleration in eye growth is a developmental phenomenon largely independent of initial ocular biometrics. From a clinical perspective, these results underscore the importance of initiating myopia surveillance and preventive strategies at the earliest ages (≤7 years), when the eye is undergoing its most rapid phase of axial elongation. This aligns with the emerging emphasis on “pre-myopia” management—intervening during the pre-myopic phase when axial growth is most aggressive, rather than delaying action until after myopia has already developed (9, 10). Our data support shifting the focus of myopia control programs toward younger children, as delaying intervention until school age may miss the period of highest susceptibility to rapid axial elongation.

Clinical research specifically targeting the pre-myopia period remains scarce. Beyond the studies by Xiang et al. (7) and Hu et al. (8), several investigations have focused on identifying risk factors that predict the onset of myopia. The CLEERE study (11) followed more than 4,000 ethnically diverse U.S. children aged 6–13 years with spherical equivalent ≤ + 3.00 D who remained emmetropic until age 14. The results showed that younger age, female sex, and Asian or Hispanic ethnicity were all associated with a higher risk of developing myopia. A study from Wenzhou Medical University (12) found that over a two-year period, 27.6% of initially non-myopic children developed myopia; key predictors of incident myopia included lower positive relative accommodation (PRA; OR = 1.4 per 1 D decrease), less hyperopic spherical equivalent (SE; OR = 11.5 per 1 D decrease), longer axial length (OR = 2.3 per 1 mm increase), and female sex (OR = 2.2). While these studies identified important risk factors for myopia onset, they primarily characterized risk at a single time point. Our findings extend this literature by demonstrating that axial elongation itself decelerates progressively with age—a pattern that was evident even after adjusting for baseline AL and baseline SER. This suggests that younger children not only face a higher risk of developing myopia, but also experience a more rapid physiological rate of eye growth during the pre-myopic phase. Both the CLEERE and Wenzhou studies also identified female sex as a risk factor for myopia onset.

Current interventions targeting the progression from pre-myopia to myopia include specially designed spectacle lenses (13), low-intensity repeated red light (LRRL) therapy (14), increased outdoor activities (15), and low-dose atropine (16). Compared with the extensive literature on myopia control after onset (6), research on pre-myopia intervention remains relatively limited. The randomized controlled trial by Zhang et al. (13) demonstrated that highly aspherical lenslet (HAL) lenses can significantly slow axial elongation in low-hyperopic children who wear them for more than 30 h per week. In the study by Liu et al. (17), pre-myopic children receiving repeated low-level red light (RLRL) treatment showed an axial length change of 0.145 ± 0.175 mm, compared with 0.292 ± 0.128 mm in the control group (*p* < 0.001). Regarding low-dose atropine, the Yam LAMP2 study (18) reported that the 2-year cumulative incidence of myopia was significantly lower in the 0.05% atropine group (28.4%) than in the placebo group (53.0%), whereas 0.01% atropine was ineffective. Collectively, these intervention studies suggest that the pre-myopic window represents a modifiable period for slowing eye growth. Our finding that axial elongation is most rapid in the youngest children—before any observable deceleration—implies that initiating these preventive strategies at the earliest ages (≤7 years) may offer the greatest potential benefit. Younger children with faster baseline axial growth rates may therefore represent *a priori*ty target group for future preventive trials.

Several limitations should be acknowledged. First, the retrospective single-center design and modest sample size (*n* = 53, with only 2 children in the 10-year group) limit statistical power and generalizability; no a priori sample size calculation or power analysis was performed. Second, although we adjusted for baseline axial length, baseline spherical equivalent refraction, and sex in the regression models, other established risk factors—including parental myopia, near-work duration, outdoor exposure, screen time, corneal curvature, and history of myopia-control interventions—were not measured or controlled for. Consequently, this study should be considered exploratory, and the independent effect of age on axial elongation cannot be fully disentangled from these unmeasured environmental and familial confounders. Third, the *post hoc* dichotomization of age into 6–7 years and 8–9 years was data-driven based on the observed pattern of non-significant adjacent-group differences; this approach may introduce bias and should be interpreted cautiously. Fourth, sex was included as a covariate in the continuous-age model but not in the primary categorical ANCOVA; moreover, the sample size within each age–sex stratum was too small to permit reliable examination of sex-specific age effects. Fifth, cycloplegia was achieved with 0.5% tropicamide rather than cyclopentolate, which is considered the gold standard for pediatric refractive studies; although we employed a standardized six-drop protocol with confirmed absence of the light reflex before refraction, residual accommodation may still have occurred in some children, potentially affecting the precise classification of pre-myopia versus myopia. Sixth, the study population was highly selected—all children had already progressed from pre-myopia to myopia within 1 year—so these findings cannot be extrapolated to the general pre-myopic population or used to predict the probability of myopia onset. Finally, axial length was measured at only two time points (baseline and 1 year follow-up), precluding assessment of seasonal fluctuations or non-linear growth trajectories within the conversion year. Larger prospective multicenter studies with comprehensive assessment of environmental and familial risk factors, standardized cycloplegic protocols, and more frequent longitudinal measurements are warranted to confirm these findings and clarify the causal role of age in axial elongation during the pre-myopic phase.

## 5 Conclusion

Pre-myopia is an important concept in myopia prevention and control. Myopia prevention and control should target the transition from myopia onset back to the premyopia stage. Among premyopic children, age is a key reference factor: the younger the age at which myopia occurs, the greater the annual axial elongation during the transition from pre-myopia to myopia, demonstrating an age-dependent decreasing effect. Younger children with faster axial elongation during the pre-myopic phase may represent a priority target group for future preventive trials. Future randomized studies are needed to determine whether early intervention in this high-risk window can effectively delay myopia onset.

## Data Availability

The original contributions presented in the study are included in the article/supplementary material, further inquiries can be directed to the corresponding author.
